# Development and validation of an instrument to measure pediatric nurses' adherence to ethical codes

**DOI:** 10.1186/s12910-022-00753-4

**Published:** 2022-02-25

**Authors:** Raziyeh Beykmirza, Lida Nikfarid, Reza Negarandeh, Naeimeh Sarkhani, Mahboube Moradi Cherati

**Affiliations:** 1 Nursing and Midwifery Care Research Center, School of Nursing and Midwifery, Tehran University of Medical Sciences, Tehran, Iran; 2 Department of Pediatrics and Reproductive Health, School of Nursing and Midwifery, Shahid Beheshti University of Medical Sciences, Tehran, Iran; 3 Markaz Tebi Koodakan Hospital, Tehran University of Medical Sciences, Tehran, Iran

**Keywords:** Nursing, Ethical codes, Pediatric, Adherence, Psychometrics

## Abstract

**Background and aim:**

The nature of pediatric settings may encounter nurses with more complicated ethical issues. A code of ethics guides nurses to act and decide ethically as a profession. Also, there is always a need to evaluate amount nurses adhere to this code of ethics, using valid and reliable instruments. This study aimed to develop a questionnaire and assess its psychometric properties to measure pediatric nurses' adherence to the code of ethics.

**Methods:**

In this methodological research study, firstly, the questionnaire was developed based on an extensive review of the related literature and the theoretical framework of nursing ethics. A panel of experts (n = 12) reviewed the preliminary questionnaire qualitatively and quantitatively (using CVI and CVR). A conveniently selected sample of 156 nurses working in pediatric wards in three hospitals filled out the questionnaire. The psychometric process included determining sample size and data adequacy using KMO and Bartlette's test of sphericity; exploratory factor analysis (principal component method with Promax rotation); item analysis; and Cronbach's alpha coefficient. Also, the Interclass Correlation Index (ICC) value was determined using a two-week interval test–retest method on 30 eligible nurses.

**Results:**

The CVI and CVR for the entire questionnaire were 0.85 and 0.78, respectively. The CVI and CVR of all items were reported higher than 0.59 and 0.8, respectively. Cronbach's alpha of the 28-items instrument was 0.92. Extracted six factors explained 65.31% of the total variance, and the values of the item correlations with the total questionnaire showed good internal consistency (0.52 to 0.90). The items of each factor were evaluated to determine the values they represent. Accordingly, the factors were named beneficence, nonmaleficence, human dignity, autonomy, informed consent, and honesty. The ICC value was 0.99.

**Conclusions:**

The developed instrument is acceptable and has good reliability and validity. It can be used to assess the amount of pediatric nurses' adherence to the code of ethics by managers, teachers, and researchers.

## Introduction

Provision of high-quality care is the essential responsibility of nurses [1]. Undoubtedly, nursing is a profession facing various ethical challenges in different fields [2]. Recently, reporting ethical challenges are increasing in the clinical care environment due to advancements in medical technology, changes in structure and functions of societies and related organizations, and increased costs of healthcare services [3]. Like any profession, nursing needs guidance for its specific actions and decisions, and the shared values as code of ethics in nursing address this need. According to these values, the ethical behaviors of nurses are evaluated [4, 5]. To make the right decisions and make proper judgments in complicated clinical practice, nurses should be familiar with the code of ethics approved and adopted for their society [4]. The code of ethics provides the opportunity to share the profession's values and responsibilities in nursing [1, 4]. Adherence of nurses to the code of ethics improves professional advancements, such as the feeling of competence through building trustful relationships with patients [2].

The International Council of Nurses (ICN) developed the first international nursing code of ethics in 1953, which described the ethical norms of nursing actions in practice, management, research, and education [2, 3]. Ethical codes need to be congruent with culture, religion, and the dominant belief system of a society. Accordingly, many communities have revised and designed their code of ethics to adapt to their culture [4]. In 1993, a center for research on medical ethics was established in the Ministry of Health and Medical Education as a first step toward developing a code of ethics according to Iranian society, which eventually resulted in developing testament of the National Code of Ethics for nursing education, management, health, research, and clinical settings in 2011 [2]. It consists of 12 values and classifies 71 provisions [1]. The values of the testament are Respecting the patient/client and preserving human dignity; Altruism and sympathy; Devotion to professional obligations; Accountability, responsibility, and conscience; Justice in services; Commitment to honesty and loyalty; Maintaining patient’s privacy, and commitment to confidentiality and trust; Continuous improvement of scientific and practical competence; Promote the awareness of professional rules and ethical guidelines, and respecting them; Mutual respect and appropriate communication with other health care providers; Respecting the autonomy of the patient/client; and Compassion and kindness [1, 4].

Ever since researchers more noticed the application of the code of ethics [2, 3, 5–15]. Most of the studies aimed to explore nurses, patients, managers, and nursing students' perceptions regarding nurses' adherence to and performance of the code of ethics in various settings. The instruments were not the same in these studies, and all were researcher-made without a precise explanation of the development process. As noticed in some of the studies with a more detailed report, each item of the questionnaire represented one of the provisions of the National Code of Ethics for Nursing (2009–2011) written in the form of behavior of nurses presenting adherence of them to that provision [2, 6, 15]. In summary, there is a lack of valid instruments psychometrically evaluated through methodological studies to evaluate nurses' adherence to the code of ethics in Iran.

Besides, few studies are related to the adherence to the code of ethics in pediatric clinical settings. The population of children is highly exposed to the non-adherence of healthcare providers to the code of ethics due to their vulnerability, inability to make decisions for themselves, and lack of capacity to understand the rationale of interventions done by others on them. Ethical principles such as authority, human dignity, privacy, and confidentiality can easily be compromised in pediatric settings [16–18]. The systematic review of the literature conducted by Pokorski and Barton in 2020 revealed that although the code of ethics is established to guide nurses in research and practice, they are not adhered to by researchers in the field of pediatric nursing [19]. In the study of Beykmirza and colleagues (2019), it was reported that Nurses reported significantly higher levels of ethics than mothers of children with cancer [20].

So, it is necessary to examine and evaluate adherence to ethical principles in the pediatric setting in a more precise way by developing specific tools for this population. According to researchers' knowledge, only one study was conducted on the pediatric population children and their parents, which used a modified form of the other studies' instrument to assess nurses' adherence to ethical codes in children oncologic patients [20]. Given the need for validated tools for evaluating and comparing viewpoints of the unit of a child- family, and nurses in pediatric nurses, this study aimed to develop and evaluate psychometric properties of an instrument to evaluate adherence of pediatric nurses to the code of ethics.

## Materials and methods

### Design of the study

The process of developing and evaluating an instrument or determining its psychometric properties is an approach that focuses on related theory and the development of measurement tools through research [21]. This methodological research study had two stages. First, we defined the behaviors through the literature review. Then, we tested the validity and reliability of the instrument.

### Derivation of items

In this stage, to make the items, scientific databases were searched, including ProQuest, PubMed, Scopus, Google Scholar, and Elsevier. Some of the main keywords were "National Code of Ethics for Nurses in Iran", “ethical codes,” “nurse,” and “ethical code adherence”. The final selected articles focus on the process of the development of the National Code of Ethics for Nurses in Iran [1, 4, 22], or the reports of the development of instruments to assess nurses' adherence to ethical codes [2, 3, 8, 23]. The preliminary version of the instrument was developed using a deductive method with the contents of the National Code of Ethics for Nursing in Iran (2009–2011), and related instruments. Thus, the first developed instrument included a description of behaviors that show nurses' adherence to the provisions of the part of "Nurses and clinical practice" in pediatric wards. The items were written as statements scored through a 5-point Likert scale as never (0), seldom (1), occasionally (2), often (3), and always (4).

### Content and face validity

Content validity assesses the extent to which an instrument includes all of the main dimensions relevant to the examined construct. Judgment is the basis of content validity [21, 24, 25]. Because of the possibility of ignoring some other main items and lack of comprehensiveness and adequacy of items [21, 26], the instrument was first evaluated qualitatively by a panel of experts (n = 12) selected through a convenience method. The experts provided their opinions about the code of ethics, pediatric ethical considerations, and instrument psychometrics as adequacy, appropriateness, coverage, the grammar of the items, and scoring method.

The initial instrument was sent to the same panel of experts to measure a content validity ratio (CVR) and content validity index (CVI); they were asked to determine whether an item was necessary for a constructor not by choosing “not necessary,” “useful but not essential,” or “essential” for each item and scoring them from one to three, respectively.

The formula of the CVR for each item was CVR =  (Ne − N/2)/(N/2), in which the Ne is the number of panelists indicating "essential," and N is the total number of experts. The CVR value is varied between 1 (perfect agreement) and − 1 (perfect disagreement). In the next stage, the CVR value was compared to a table of critical CVR values (the Lawshe table), which determined whether an item was kept or not [24, 27].

One other method to evaluate content validity is CVI. That is a quantitative description of the extent to which a panel of experts agrees on the relevance of items in a new-made tool. In this study, the CVI was calculated as an item-level content validity index (I-CVI) and a scale-level content validity index (S-CVI) [21, 28]. The I-CVI shows the proportion of agreement on the relevancy of each item, and its score is between 0 and 1. In this study, the panel of experts was requested to rate the relevance of each item, on a four-point scale, as “not relevant,” “somewhat relevant,” “quite relevant,” and “very relevant” from one to four, respectively [28]. Then, the report of four ordinal answer options was put into two categories (one and two) of not relevant and relevant (three and four). To measure S-CVI, the average of the I-CVIs was calculated by dividing the sum of I-CVIs by the total number of items (n = 28).

One other psychometric property is face validity. In this study, five experts who were not in the previous expert panel were asked to evaluate items in terms of simplicity, fluency, and comprehension [24]. Their opinions were for each item rated on a five-point Likert scale as completely important (5), important (4), moderately important (3), slightly important (2), and not important (1). The ratio of experts who had selected 4 and 5 for each item, the sum of scores given to each item, and the average score of each item were calculated. The items with an impact factor score equal to or above 1.5 are considered as acceptable. Also, for conducting qualitative face validity, based on a purposeful sampling, ten nurses of the target group were asked to give their opinions on the level of difficulty and comprehensibility of the terms, relatedness, and appropriateness of the items, as well as any ambiguity, found in the terms or phrases.

### Construct validity

Through conducting a construct validity, the extent to which items can explain the main concepts of the study (the values of the National Code of Ethics) is determined [24, 25]. Item and exploratory factor analysis were used to describe, classify, and summarize data. According to the literature, the sample size for factor analysis should be at least five times the initial number of items [29]; therefore, the calculated sample size was 160. This calculation included a 10% probability of sample loss. A random drawing strategy was used as a sampling method to obtain the nurses' names who had the inclusion criteria and give a number to each of them. The two inclusion criteria included having at least one year of experience working as a pediatric nurse in one of the three educational pediatric and willingness to participate in the study.

The developed instrument consisted of two parts; the first included demographic information (age, gender, nursing experience with children, higher level of education, having children, marital situation, and participation in workshops related to nursing ethics). The second part included 28 behaviors representative of nurses' adherence to the code of ethics in pediatric fields.

After obtaining informed consent, the nurses filled out the questionnaire. The first researcher gave the questionnaire to each nurse. The incomplete questionnaires would be excluded.

### Reliability

Reliability is the degree to which participants maintain their opinion in a sample of repetitive dimensions. In this study, the Interclass Correlation Index (ICC) value was determined using a 2-week test–retest method for a group of 30 nurses. The minimum rate of ICC was considered 0.75. To test the instrument's internal consistency, Cronbach’s alpha coefficients were used, and the minimum value of 0.7 knew as acceptable. The conceptual compatibility of items of an instrument is measured through internal consistency [25].

### Data analysis

Data were analyzed using the SPSS Version 23 (IBM Corp, Armonk, NY). To describe quantitative and categorical variables, mean (standard deviation) and frequency were used, respectively. The psychometric process included Kaiser–Meyer–Olkin's (KMO) measure of sampling adequacy and Bartlett’s test of sphericity to assess data adequacy for exploratory factor analysis. Scree plot, principal component analysis (PCA), and Promax rotation were used to reduce data. Items were placed in factors based on their correlation with that factors [25, 30].

## Findings

Three items of the preliminary questionnaire were deleted while adding to other items, according to the opinions of the expert panel. In addition, necessary corrections were made for other items. At this point, the instrument consisted of 28 items. The impact factor score of these items was all above 1.5. The target group of nurses for determining the face validity of the tool confirmed the comprehensibility of the terms, relatedness, and appropriateness of the items.

The CVI and CVR for the total tool were 0.85 and 0.78. respectively. Item CVR scores were more than 0.59, and item CVI scores were more than 0.8. The expert panel approved all 28 items in terms of face validity. According to the values of item effect size (above 1.5), the items did not need any modification.

The total sample size was 158. Two nurses did not participate in the study. In this stage, 15.8% (n = 25) of nurses were male, and the rest were female (n = 133). The mean age was 31.83 ± 6.41. The mean years of experience were 8.12 ± 5.76 years. In addition, 72.8% of nurses had 0 or 1 child. The majority of nurses (75.9%) had bachelor’s degrees. 57.6% (n = 91) had participated in workshops related to nursing ethics.

KMO test value of 0.898 and significance of Bartlett's Test of Sphericity (χ^2^ = 2479.005, df = 378, *p* value < 0.000) confirmed sample adequacy for conducting an exploratory factor analysis. The KMO value indicates zero-order correlation matrix of the elements is larger than the matrix. Factors can be extracted from the matrix. The significant value of Bartlett's test of sphericity showed that the data correlation matrix in the sample is not zero, and therefore, factorization is justifiable.

**Fig. 1:**
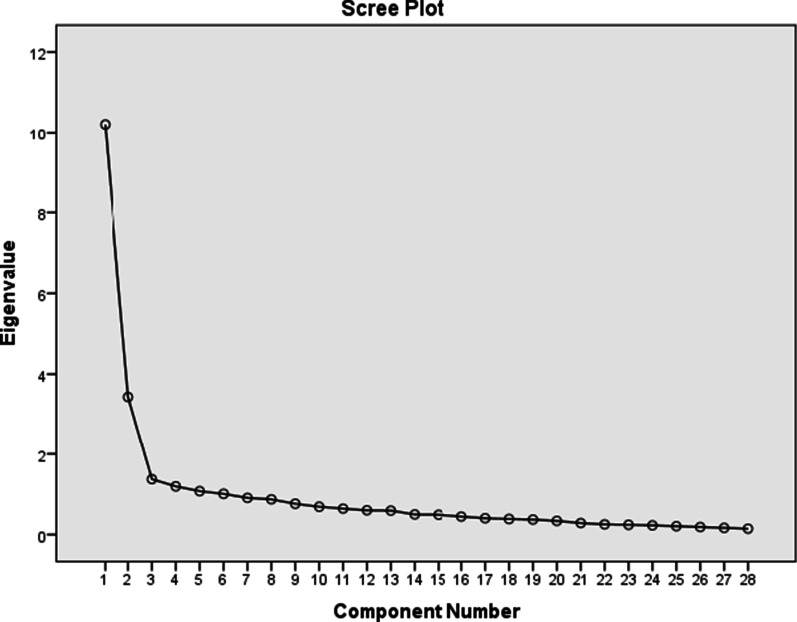
Scree plot for factors of the instrument

The latent factors were determined using a scree diagram (Fig. 1), eigenvalues, and percentage of variance explained by each factor. Applying principal component analysis, the underlying factor structure of the 28-item instrument was determined. After Promax rotation, the distribution of cumulative variance showed that six factors consisting of all the 28 items have 65.31% of the total variance of nurses' adherence to the code of ethics in pediatric wards (Table 1). All the items had loading values above 0.5, ranging from 0.526 to 0.796, meaning that all have a common variance with each other. The extracted factors were named "beneficial", "nonmaleficence", "human dignity", "autonomy", "informed consent" and "honesty", respectively. The final component rotated matrix of the instrument, and their items' loadings are presented in Table 2.

**Table 1:** The results of principle component analysis, factors and variances explained

	Initial Eigenvalues			Extraction of squared loadings			Rotation sum of squared
	Total	% of variance	Cumulative %	Total	% of variance	Cumulative %	Total
1	10.205	36.447	36.447	10.205	36.447	36.447	8.027
2	3.433	12.262	48.709	3.433	12.262	48.709	7.401
3	1.372	4.900	53.608	1.372	4.900	53.608	8.079
4	1.194	4.263	57.871	1.194	4.263	57.871	3.782
5	1.075	3.838	61.709	1.075	3.838	61.709	2.769
6	1.009	3.602	65.311	1.009	3.602	65.311	2.065

**Table 2:** Final component rotated matrix of the instrument and their items' loading

Items	Components					
	1	2	3	4	5	6
1. I don't discriminate children in providing qualified care to them	0.901					
2. I do not provide personal and health-related information to anyone without their consent	0.892					
3. I use the child file information only for therapeutic purposes	0.731					
4. I resolve any objections or problems of children and families within my authority or inform the head of the ward	0.715					
5. I always try to gain the trust of children and their families	0.554					
6. I don’t discriminate between patients according to their social, economic, cultural, religion and nation characteristics	0.508					
7. I teach children and families to empower them in terms of the process of living with a disease		0.932				
8. I precisely monitor child to prevent any physical trauma (such as fall) during hospitalization		0.844				
9. In performing nursing care, I fully follow the principles of aseptic to prevent infection		0.780				
10. I precisely adhere the medication administration rules to prevent any error		0.629				
11. I precisely listen to children and their parents			0.902			
12. I respect every cultural and religious believers of children and their families			0.857			
13. I respect child's privacy in every intervention and encounter			0.565			
14. I deal with children based on their developmental age			0.533			
15. I speak politely with children and their parents			0.482			
16. I emotionally support children and families before, during and after every procedure			0.471			
17. I ask the name of child to be sure about right patient before any intervention			0.440			
18. I try to meet the needs of the children and their families as soon as possible and within my authority			0.333			
19. I encourage families to decide about the health- related issues of their children				0.802		
20. As much as possible, I encourage older children to make decisions about their health				0.702		
21. As much as possible, I encourage the families to make decisions about the health issues of their children				0.657		
22. When obtaining informed consent for diagnostic and therapeutic interventions, I provide the family with complete information about the right to knowingly reject or accept any procedure				0.542		
23. Before each intervention, I explain the procedure and the reason for doing it in simple language to the child and parents					0.819	
24. I explain the required care information from the time of admission to discharge and then in simple language to the children and their families					0.725	
25. I explain the effect and side effects of any medication to the children and families					0.709	
26. Before providing care, I introduce myself to the children and their families by name and mention the duties						0.739
27. I answer questions honestly with the child and family about health and illness issues						0.665
28. In nursing of children, I am honest with them						0.456

Extraction method: principal component analysis. Rotation method: Promax with Kaiser normalization (Rotation converged in 8 iterations)

For the final 28-item instrument, the total Cronbach's alpha was 0.92, which means an excellent internal consistency. The Cronbach's alpha for the factors of 1 to 6 were 0.90, 0.88, 0.77, 0.75, 0.75 and 0.52, respectively (Table 3). Besides, the obtained ICC value was 0.96 for the total instrument (Table 4).

**Table 3:** Cronbach's alpha amounts and ICC for the instrument and its factors

Domains	Number of items	Cronbach's alpha	*p* value
Beneficence	6	0.90	< 000
Nonmaleficence	4	0.88	< 000
Human dignity	8	0.77	< 000
Autonomy	4	0.75	< 000
Informed consent	3	0.75	< 000
Honesty	3	0.52	< 000
Total	28	0.92	< 000

**Table 4:** Results of ICC using single-rating, absolute-agreement, 2-way random-effects model

Single measures	Interclass correlation	95% confidence interval		F test with true value 0			
		Lower bound	Upper bound	Value	df1	df2	Sig
	0.966	0.465	0.994	148.634	9	9	0.000

## Discussion

The developed instrument in the present study is distinct as specified for the pediatric settings. It had a comprehensive development process and psychometric evaluation; its dimensions' names are consistent with the ethical principles in nursing.

All the 28 items were retained based on the face validity results. This confirmed instrument was assessed for construct validity through exploratory content validity. According to the results of Kaiser–Mayer–Olkin sampling indexes and Bartlett's test, the conduction of exploratory factor analysis was considered justifiable. Factor analysis is allowed if the level of KMO is above 0.5.

Exploratory factor analysis revealed six factors for the developed instrument. In some factors, the content of all items was not consistent despite the high collation. We named each factor according to the ethical principle with the most items.

The first factor had six items, called beneficence. Beneficence is one of the main principles in nursing ethics [4]. The content of the items of this factor included moral values such as justice, respect for privacy, confidentiality, and benevolence for the patient. Beneficence is a central principle in medical and nursing ethics and includes the obligation of the nurse to observe the ethical principles, namely altruism, benevolence, confidentiality, and justice in their professional dealings [31, 32].

The first factor included items related to the principle of justice (two items), privacy (one item), confidentiality (one item), and supporting children and their families (one item). The small number of items and the lack of differentiation of ethical principles in the provisions of the Code of Ethics for Nurses can explain this result. Therefore, to further differentiate ethical principles in pediatric wards, it is necessary to design more accurate tools.

The second factor was called nonmaleficence that addresses behaviors that protect the children and provide their safety. This moral principle also requires more sensitivity in pediatric wards. For example, studies have shown that nurses show less professional sensitivity to children's pain and that painkillers are much lower in this population than in adults [33, 34]. Observing the safety and protection of the children from various trauma that may threaten them in the hospital is one of the essential responsibilities of pediatric nurses [35, 36]. One item of this factor deals with the need for education and empowerment of the family. Despite the inconsistency with other items of this factor, we kept it because of the importance of the concept of family-based care in pediatric nursing. Here, to further differentiate the moral behaviors related to these two principles, it is necessary to build a tool to measure the adherence to the code of ethics through methodologies. The same is true of the third factor, which had several items and referred to various moral values. Respect for human dignity, privacy, meeting needs, and proper communication are the main themes of this factor, which describe the different professional responsibilities of nurses towards children in the form of objective behaviors. Children in hospitals are more at risk of non-compliance with their human rights than adults. For a nurse to be able to provide professional care for infants and children, it is necessary to first believe in their human rights as in other age groups

The fourth factor, including four items, and the two other three-items last factors, were named autonomy, informed consent, and honestly, respectively, according to the contents of their items. These three ethical principles also need to be paid more attention to in the children, as their parents make clinical decisions for their clinical issues [37]. Older children who are still in the custody of their parents but have sufficient understanding of their health issues have the right to independence and informed consent of different content and nature than adults. The Code of Ethics for Nurses (2011) has several general provisions that result in a lack of differentiation populations that do not have the legal right to make clinical decisions for themselves. We designed the questionnaire items investigating the nurses' perceptions about their adherence to the code of ethics for the family unit, including parents and children. Another study is needed to develop specified instruments for evaluating the adherence exclusively to ethical principles in dealing with infants and children regardless of parents.

The instrument's internal consistency assessed by the Cronbach alpha coefficient value equaled 0.92 for the total questionnaire and 0.52–0.90 for its dimensions. Cronbach alpha coefficient value equal to or above 0.7 is considered desirable and an indication of the internal consistency of an instrument. The ICC for the total instrument was 0.99; values equal to or above 0.4 show a tool's stability and reliability.

## Conclusion

In summary, the developed instrument to evaluate pediatric nurses' adherence to the Code of Ethics for Nurses (2011) is acceptable and has good reliability and validity. Pediatric nurse managers, researchers, and teachers can use this instrument for clinical audits, research, and education. As a limitation, we developed the questionnaire only based on literature review and based on the provisions of the Code of Ethics for Nurses (2011), so ethical principles are not separate, and in one factor, there may be items related to more than one principle. Accordingly, the questionnaire can overview the observance of ethical principles. Interpreting each item's score can also help researchers find ethical behaviors that are less common among nurses in pediatric wards.

## Data Availability

The datasets generated and/or analysed during the current study are not publicly available due to limitations of ethical approval involving the patient data and anonymity but are available from the corresponding author on reasonable request.
